# Pyrotag Sequencing of the Gut Microbiota of the Cockroach *Shelfordella lateralis* Reveals a Highly Dynamic Core but Only Limited Effects of Diet on Community Structure

**DOI:** 10.1371/journal.pone.0085861

**Published:** 2014-01-15

**Authors:** Christine Schauer, Claire Thompson, Andreas Brune

**Affiliations:** 1 Department of Biogeochemistry, Max Planck Institute for Terrestrial Microbiology, Marburg, Germany; 2 LOEWE Center for Synthetic Microbiology, SYNMIKRO, Philipps-Universität Marburg, Marburg, Germany; 3 School of Molecular Bioscience, The University of Sydney, Sydney, Australia; University of Freiburg, Germany

## Abstract

Although blattid cockroaches and termites share a common ancestor, their diets are distinctly different. While termites consume a highly specialized diet of lignocellulose, cockroaches are omnivorous and opportunistic feeders. The role of the termite gut microbiota has been studied intensively, but little is known about the cockroach gut microbiota and its function in digestion and nutrition, particularly the adaptation to different diets. Our analyses of the bacterial gut microbiota of the blattid cockroach *Shelfordella lateralis* combining terminal restriction fragment length polymorphism of their 16S rRNA genes with physiological parameters (microbial metabolites, hydrogen and methane emission) indicated substantial variation between individuals but failed to identify any diet-related response. Subsequent deep-sequencing of the 16S rRNA genes of the colonic gut microbiota of *S. lateralis* fed either a high- or a low-fiber diet confirmed the absence of bacterial taxa that responded to diet. Instead, we found a small number of abundant phylotypes that were consistently present in all samples and made up half of the community in both diet groups. They varied strongly in abundance between individual samples at the genus but not at the family level. The remaining phylotypes were inconsistently present among replicate batches. Our findings suggest that *S. lateralis* harbors a highly dynamic core gut microbiota that is maintained even after fundamental dietary shifts, and that any dietary effects on the gut community are likely to be masked by strong individual variations.

## Introduction

Blattid cockroaches are the closest relatives of termites, derived from a common, presumably omnivorous ancestor [1]. While termites have acquired an intestinal microbiota that provided the ability to digest an entirely lignocellulosic diet, most extant cockroaches remained generalists that scavenge a broad range of more easily digestible substances from their environment. Our previous analysis of the bacterial community in the colon of the cockroach *Shelfordella lateralis*, a member of the Blattidae, the sister family of termites [1], revealed that many of its gut bacteria belong to the same lineages as those present in termites, suggesting that the composition of the gut microbiota reflects the close phylogenetic relationship of its hosts [2]. The shared presence of several bacterial lineages common to termites was reported also in a preliminary analysis of the gut microbiota of *Periplaneta americana* [3], a close relative of *S. lateralis*.

Although the intestinal microbial communities of cockroaches have been studied only in a few blattid species [2], [3], it is obvious that the gut microbiota fundamentally differs from that of termites. Besides the complete absence of cellulolytic flagellates, cockroaches appear to lack the bacterial phyla *Spirochaetes* and *Fibrobacteres* [2], which are abundantly represented in termites and whose members have been implicated in fiber digestion [4], [5]. It is likely that differences in the composition of the gut microbiota of termites and cockroaches reflect adaptations to their respective diets.

Although most cockroaches are generalists, the composition of their diet plays an important role in development [6]–[9]. Nutrient-poor diets have been shown to cause significant physiological stress, increasing mortality [10], [11], extending development time [12], altering reproductive capacity [11], [13], and changing foraging behavior [14]. In contrast to termites, where the role of the gut microbiota in digestion and nutrition has been studied intensively [15], [16], our understanding of symbiotic digestion in cockroaches is quite superficial. Gut microorganisms of cockroaches break down dietary substances, supply volatile fatty acids, and contribute to both the development and nutritional status of their host [17], [18]. It has been shown that the reduction of the bacterial community by antibiotic treatment reduces body weight and impedes development of *Periplaneta americana* [17], [19]. However, the response of the gut microbiota to changes in diet has not been studied.

In cockroaches, the most important site for symbiotic digestion is the colon. In *S. lateralis*, the colon has the highest diversity and density of bacteria of all gut compartments [2]. In *P. americana*, the colon is the site for cellulose and hemicellulose degradation [20]. However, previous studies that have examined the impact of dietary shifts have mostly focused on microbial activities rather than on changes in diversity and community structure of the colonic microbiota. For example, high-fiber diets have been shown to increase methane production and volatile fatty acid concentrations in *P. americana* [21], [22].

Here, we investigated the response of the *S. lateralis* gut microbiota to different diets varying in fiber and protein content. Preliminary analyses using terminal-restriction fragment length polymorphism (T-RFLP) and methane emissions of individual cockroaches revealed substantial variations in community structure but failed to identify any diet-responsive bacterial groups. Therefore, we used deep sequencing of 16S rRNA genes to increase both sampling depth and phylogenetic resolution, focusing on batches of cockroaches fed either a high- or a low-fiber diet.

## Materials and Methods

### Cockroaches and diet

*Shelfordella lateralis* was obtained from a commercial breeder (J. Bernhard, Helbigsdorf, Germany) and maintained in a temperature controlled chamber at 25°C with 50% humidity, as previously described [2]. Cockroaches were fed one of four diets: a balanced diet of chicken feed (CF) (4% fiber, 16% protein; Gold Plus, Versele-Laga, Deinze, Belgium), a high-protein diet of soy meal (S) (7% fiber, 43% protein; Ruppersberg, Cölbe, Germany), a fiber-rich diet of wheat bran (B), (36% fiber, 15% protein; Spielberger, Brackenheim, Germany), or a fiber-rich diet of wheat bran supplemented with 30% cellulose powder (BC) (Sigma-Aldrich, Steinheim). Food and water were provided *ad libitum*. For each diet, two replicate batches were established and maintained on each diet for 3 months, a period sufficient for cockroaches to go through at least two developmental stages. After three months, the gut was extracted from adult cockroaches (the individual times after the final molt were not recorded), the gut compartments were weighed individually, and colons were frozen at −20°C for further use. Significant differences between gut weights were determined by the Kruskal-Wallis nonparametric analysis of variance (ANOVA) in R (version 2.10) [23].

### Microbial cell counts

Guts were dissected, and the contents of each gut compartment were squeezed out gently with a pair of forceps. The material was weighed and diluted 1∶100 in phosphate-buffered saline (pH 7.2), and microbial cell densities were measured as previously described [24]. Briefly, the suspensions were stained with 4′,6-diamidino-2-phenylindole (DAPI) and applied to polycarbonate filters (0.2 µm, GTTP, Millipore) using a vacuum pump. For quantification, each filter was divided into quarters, and five fields per quarter were counted using a fluorescence microscope (Axiophot, Zeiss).

### Microbial metabolites

Metabolites were quantified by ion-exclusion chromatography using an HPLC system equipped with a Grom Resin IEX column (8 µm, 250×4.6 mm i.d., Grom, Rottenburg, Germany) and a refractive index detector (RID-10A, Shimadzu) with a mobile phase of 5 mM H_2_SO_4_ and a column temperature of 60°C. Peak identity was verified using external standards. Samples for HPLC were prepared as previously described in Schauer *et al.* [2]. Briefly, individual colons were homogenized in 200 µl water and centrifuged for 10 min at 20,000×*g*. The supernatant was acidified with one volume of 100 mM H_2_SO_4_ and filtered (0.2 µm, ReZist, Whatman). Significant differences in concentrations of gut metabolites were determined by the Kruskal-Wallis nonparametric analysis of variance (ANOVA) in R (version 2.10) [23].

### Hydrogen and methane emission

Hydrogen and methane emission rates for individual cockroaches were assessed by placing each cockroach in 15 mL glass vial that were closed with a rubber stopper. Gas emissions were measured every 30 min by gas chromatography using a packed column (Porapack Q column, 80/100 mesh; 274 cm×3.18 mm) and a methanizer coupled to a flame ionization detector. Stimulation of methane emission was tested via addition of 25% hydrogen to the headspace. Hydrogen was measured by gas chromatography using a packed column (Mol Sieve 5A, 80/100 mesh; 70 cm×6.35 mm) and a reduction gas detector (RGD2, Trace Analytical, Calif., USA).

### T-RFLP analysis

DNA was extracted from the colons of individual cockroaches with phenol-chloroform extraction and precipitated with ethanol [25]. T-RFLP profiles of bacterial 16S rRNA genes were generated following the protocol of Egert *et al.* [26], both with modifications described in Schauer *et al.* [2]. Pairwise similarities between T-RFLP profiles were calculated using the Morisita-Horn index [27]. Nonmetric multidimensional scaling (NMDS) analysis was performed using R (version 2.10) and the VEGAN software package [28].

### Pyrotagsequencing of 16S rRNA genes

Colon DNA was pooled from 10 *S. lateralis* individuals of each replicate. Pyrosequencing was done as previously described [29]. Briefly, 16S rRNA genes were amplified with primers 343Fmod (TAC GGG WGG CWG CA) and 784Rmod (GGG TMT CTA ATC CBK TT) targeting the V3–V4 region. Both primers had an additional, sample-specific 6-bp barcode at the 5′ end. Adaptor ligation, subsequent amplification, and pyrosequencing (454 GS FLX with Titanium technology, Roche) were done by a commercial service (GATC Biotech, Konstanz, Germany). The sequences were classified against the manually curated reference database described by Köhler *et al.* [29], which consisted of the SILVA 102 non-redundant database amended with numerous unpublished sequences from termite and cockroach guts (Table S1). Sequences assigned to the genus *Blattabacterium*, an endosymbiont of cockroaches residing in the surrounding fat body [30], [31], were not considered part of the cockroach gut microbiota. They were removed before further analysis because the frequency of *Blattabacterium* sp. in the different samples was caused by varying amounts of residual fat body in the individual dissections. Heat maps were constructed using R (version 2.10) [23].

To assess the influence of diet on the abundance of individual phylotypes, colon DNA was obtained from adult cockroaches fed either on chicken feed (low-fiber diet) or bran-cellulose (high-fiber diet). A total of three replicate batches, each consisting of 10 individuals kept in the same box, were obtained for each diet. Bacterial community structure was assessed by pyrosequencing of 16S rRNA genes as described above, and analyzed using the mothur software [32]. After sequence processing according to the protocol described in Köhler *et al.* [29], we obtained 1,689 to 17,199 high-quality reads per sample (Table S1). Aligned sequences were clustered in phylotypes based on 97% sequence similarity, which were classified against a manually curated reference database [29]. All sequence data was deposited in the NCBI Sequence Read Archive under the project accession number SRP032804.

## Results

### Gut weight

**Figure 1:**
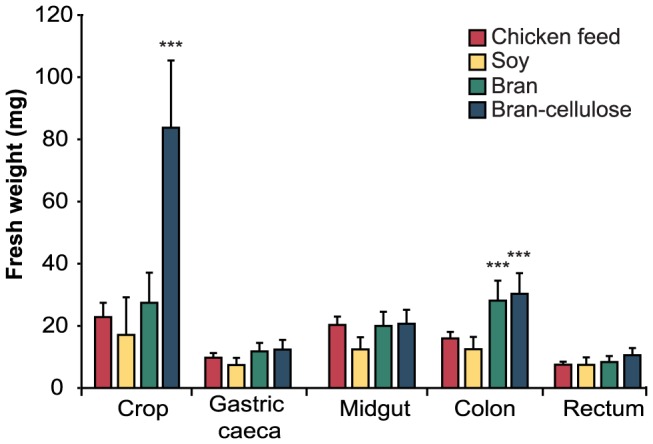
Gut compartment weight in *Shelfordella lateralis* fed different diets. Fresh weight of individual compartments is given with standard error of the mean (*n* = 30 for chicken feed, *n* = 9 for soy, *n* = 20 for bran and bran-cellulose). Asterisks indicate diet groups with significantly higher fresh weight (*p*<0.001, ANOVA).

Body weight of individual cockroaches was 597±117 mg (chicken feed; *n* = 30), 555±176 mg (soy; *n* = 9), 556±126 mg (bran; *n* = 20), and 591±129 mg (bran-cellulose; *n* = 20). While body weight was similar regardless of diet, the weight of specific gut compartments significantly differed (Figure 1). Cockroaches fed a fiber-rich diet (bran or bran-cellulose) had a significantly higher colon weight than those fed high-protein or balanced diets; crop weight was significantly higher only in individuals fed bran-cellulose.

### Microbial cell densities

**Figure 2:**
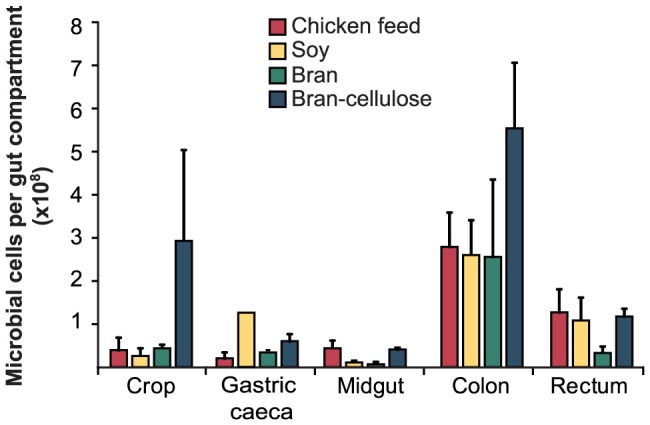
Enumeration of microbial cells within each gut compartment of individual cockroaches fed different diets. Deviations are given as standard error of the mean (*n* = 3).

In all diet groups, the largest number of microbial cells was found in the colon (Figure 2), with cell densities ranging from 9.5 to 22×10^6^ cells per mg of gut content (Table 1). The microbial cell counts in crop and colon were highest in cockroaches fed bran-cellulose (Figure 2), but since these compartments were also enlarged in bran-cellulose-fed individuals (Figure 1), the overall microbial densities in crop (not shown) and colon (Table 1) did not significantly differ from that of cockroaches from other diet groups.

**Table 1:** Microbial cell density and T-RF-based analysis of bacterial diversity within the colon of individual cockroaches fed different diets.

Diet	Chicken feed	Soy	Bran	Bran-cellulose
Microbial cell density (10^6^/mg)^1^	22.3±15.9	17.3±15.5	9.46±7.8	18.6±11.3
Number of T-RFs^2^	36±8	36±8	37±4	28±7
Shared T-RFs per diet group (per replicate batch)^3^	6 (9, 15)	5 (5, 18)	10 (13, 20)	5 (13,13)
Morisita-Horn index	0.44±0.23	0.51±0.29	0.51±0.2	0.60±0.23

All values are given as the mean with standard deviation.

^1^ Based on fresh weight (*n* = 3).

^2^ Distinct T-RFs in profiles of individual cockroaches (*n* = 6).

^3^ T-RFs represented in all individuals on the same diet (within a replicate batch).

### Microbial metabolites

**Figure 3:**
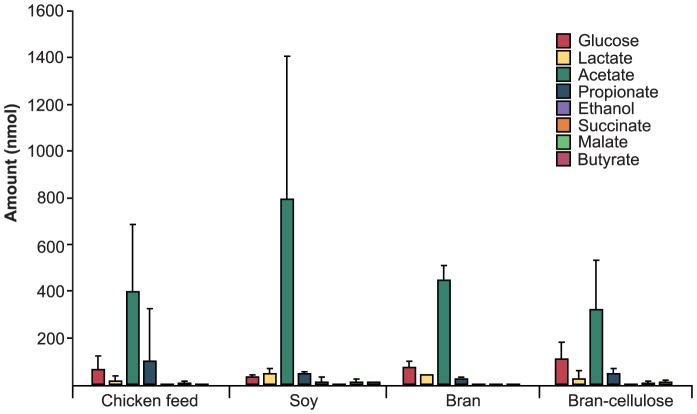
Microbial fermentation products in the colon fluid of individual cockroaches fed different diets. Deviations are given as standard error of the mean (*n* = 8). No significant differences were observed among the diet groups (*p*>0.05, ANOVA).

All cockroaches showed similar patterns of microbial fermentation products in their colon (Figure 3). There were no significant differences among diet groups. Acetate was the major metabolite in all samples, and glucose, lactate, and propionate were always present in moderate amounts. Low concentrations of succinate, malate, and butyrate were detected in a few individuals fed chicken feed, soy, and bran-cellulose.

### Hydrogen and methane emission

**Figure 4:**
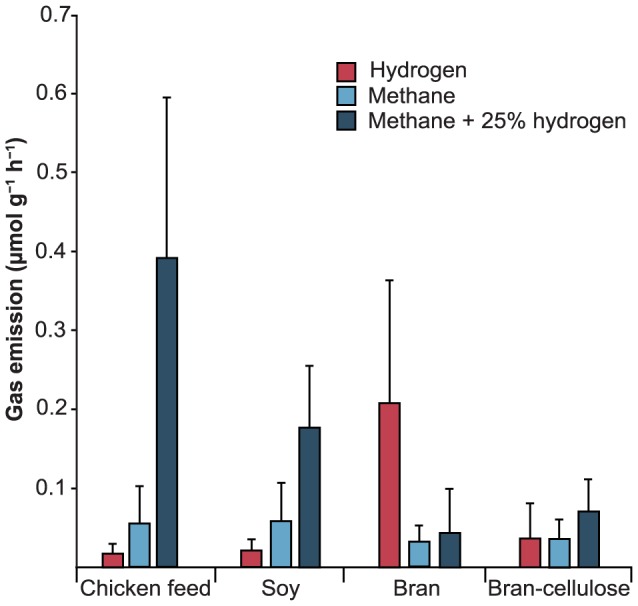
Emission of hydrogen and methane from individual cockroaches fed different diets (*n* = 11). Methane was measured both before and after stimulation by addition of hydrogen to the headspace (25% *v/v*). No significant differences were observed among the diet groups (*p*>0.05, ANOVA).

Hydrogen and methane emission rates varied strongly between individual cockroaches (Figure 4). There were no significant differences among the diet groups, with methane emission rates ranging between 0.03 to 0.05 µmol g^−1^ h^−1^. In all groups, the majority of individuals emitted methane to varying degrees. In a small number of individuals, methane production was below the detection limit (0.01 µmol g^−1^ h^−1^). However, the addition of 25% hydrogen to the headspace stimulated methane production in all individuals. This effect was more pronounced in cockroaches on a protein-rich (0.03–0.26 µmol g^−1^ h^−1^) or balanced diet (0.11–0.73 µmol g^−1^ h^−1^) than in those on fiber-rich diets (0.02–0.14 µmol g^−1^ h^−1^). Interestingly, cockroaches in the latter groups showed higher rates of hydrogen emission than those in the former groups. All individuals emitted hydrogen, but emission rates were significantly higher in bran-fed cockroaches (0.03–0.49 µmol g^−1^ h^−1^; p<0.001, ANOVA) than on those fed bran-cellulose (0.0008–0.14 µmol g^−1^ h^−1^), soy (0.004–0.06 µmol g^−1^ h^−1^), or chicken feed (0.003–0.04 µmol g^−1^ h^−1^). Respiratory CO_2_ formation varied strongly between individuals (12.6–56.0 µmol g^−1^ h^−1^) but was not correlated with diet or influenced by the addition of hydrogen.

### T-RFLP analysis of the colonic microbiota

**Figure 5:**
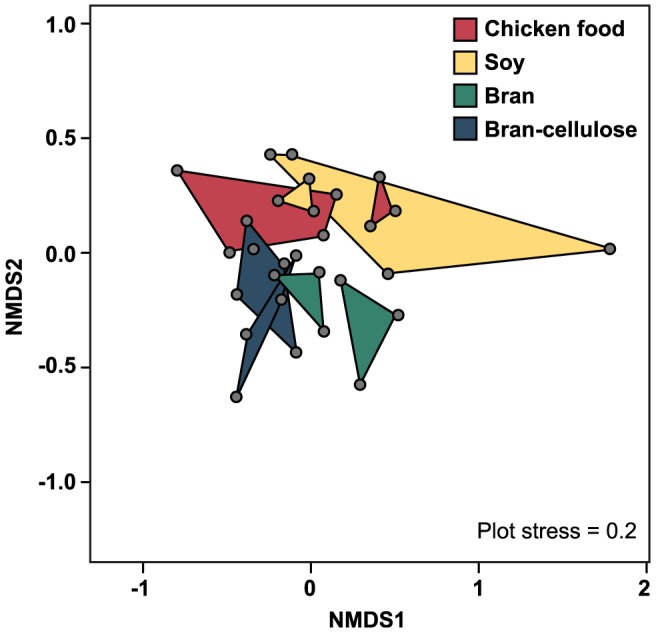
Nonmetric multidimensional scaling (NMDS) analysis of T-RFLP profiles of the colon of individual cockroaches fed different diets. Data points represent Bray-Curtis similarities; profiles of individuals from the same replicate are connected.

Diversity of the bacterial communities in the colon was assessed by T-RFLP analysis. The profiles of individual cockroaches from the four diet groups yielded a total of 126 distinct T-RFs. However, the average number of T-RFs for each diet groups was similar (Table 1), indicating that species richness was not significantly different between the diets. A pairwise comparison of T-RF patterns (Morisita-Horn index) indicated a low similarity of individuals fed the same diet, which was in agreement also with the low proportion of shared T-RFs; the number of T-RFs shared between individuals from the same replicate batch were slightly higher (Table 1). The average Morisita-Horn index of the pairwise comparison of individuals within a diet was not significantly higher than between diets (0.53±0.21), and Student's t-test did not support significant clustering of profiles from the same diet or the same replicate batch. Also Bray-Curtis similarities of the T-RF patterns showed only a marginal separation of profiles from different diets (Figure 5).

### Diversity analysis by pyrotag sequencing

**Figure 6:**
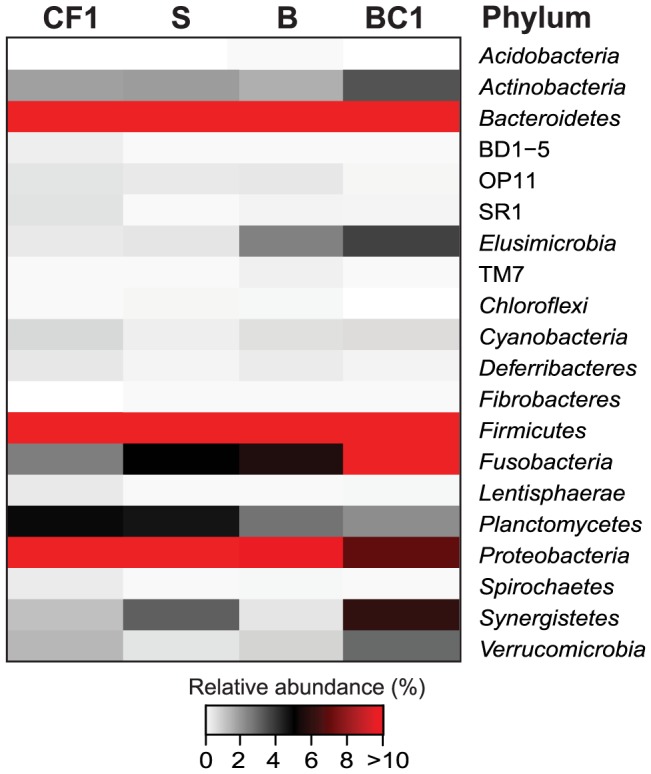
Relative abundance of pyrotag reads in samples of cockroaches fed different diets, classified at the phylum level.

To assess microbial diversity across cockroaches fed different diets, we analyzed the colonic microbiota using pyrotag sequencing of 16S rRNA genes and classified the reads against a curated reference database (Table S1). At the phylum level, there were only marginal differences between the diet groups. Of the 20 phyla represented in the entire dataset, the most abundant ones were shared among all diet groups (Figure 6). The majority of sequences fell within the *Firmicutes* (36–52% of sequences), followed by *Bacteroidetes* (13–25%), *Proteobacteria* (7–19%), *Fusobacteria* (3–15%), and *Planctomycetes* (2–5%). Sequences of candidate division TM7 were more abundant in bran- and bran-cellulose-fed animals (3–4%) than in those fed soy or chicken feed (0.4%). Members of *Synergistetes* were abundant in all diet groups (1–6%) except bran-cellulose (0.4%).

While representatives of the abundant phyla were detected already in a 16S rRNA clone library of the colon of *S. lateralis* [2], the deep sequencing approach revealed the presence of nine additional phyla. Of particular interest is the presence of *Spirochaetes* (0.03–0.3%) and *Fibrobacteres* (0.008–0.02%), albeit in low abundance. Also sequences belonging to *Acidobacteria*, *Cyanobacteria*, *Lentisphaerae*, and *Candidate divisions* BD1-5, OP11, and SR1 each represented less than 1% of the total reads; only *Verrucomicrobia* were somewhat more abundant (Table S1).

### Effect of diet on phylotype distribution

**Figure 7:**
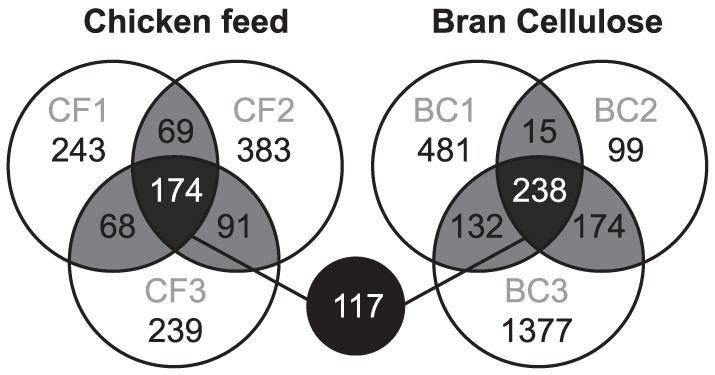
Distribution of phylotypes in replicate batches of cockroaches fed either a diet of chicken feed (7,898 sequences) or bran-cellulose (25,374 sequences). Across all replicates of both diets, 117 phylotypes were shared. Each circle represents a sample of ten colons of individuals from the same box.

We assessed the influence of diet on the abundance of individual phylotypes by comparing three replicate batches of *S. lateralis* kept either on chicken feed (low-fiber diet) or bran-cellulose (high-fiber diet). Sequences reads were grouped into phylotypes (97% sequence similarity) and then classified against the reference database. Of the 1,267 phylotypes detected in the chicken feed samples, 174 phylotypes (61% of the sequences) were shared among all replicate batches (Figure 7). Only 3 of these phylotypes (1.3% of the sequences) were unique to cockroaches fed chicken feed (i.e., not found in those fed bran-cellulose). A total of 1,921 phylotypes were detected in the bran-cellulose samples. In that case, 238 phylotypes (63% of sequences) were shared among all replicate batches, with 21 phylotypes (2.6% of all sequences) present only in this diet group, including one phylotype each of *Spirochaetaceae* and *Fibrobacteraceae*.

**Figure 8:**
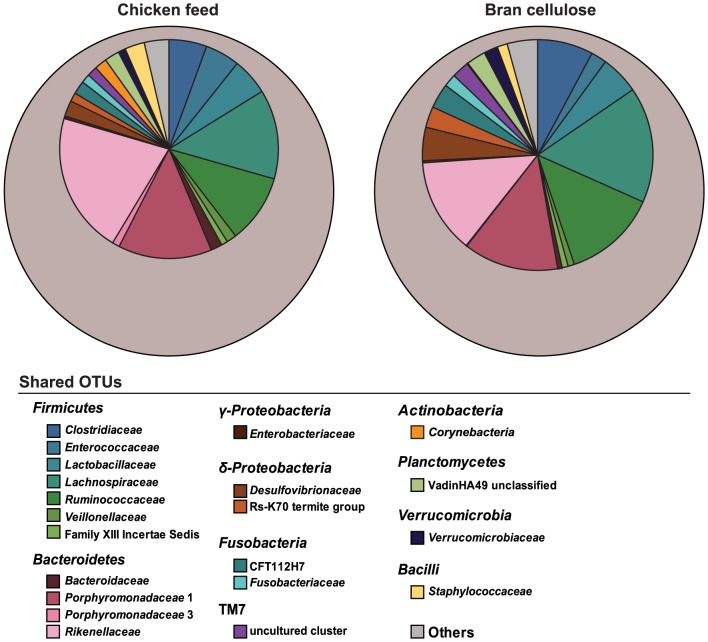
Relative abundance and family-level distribution of the 117 phylotypes shared between replicate batches of cockroaches fed either chicken feed or bran-cellulose. Values are based on the number of sequence reads for each phylotype relative to the total reads in each sample (indicated by the grey circles).

**Figure 9:**
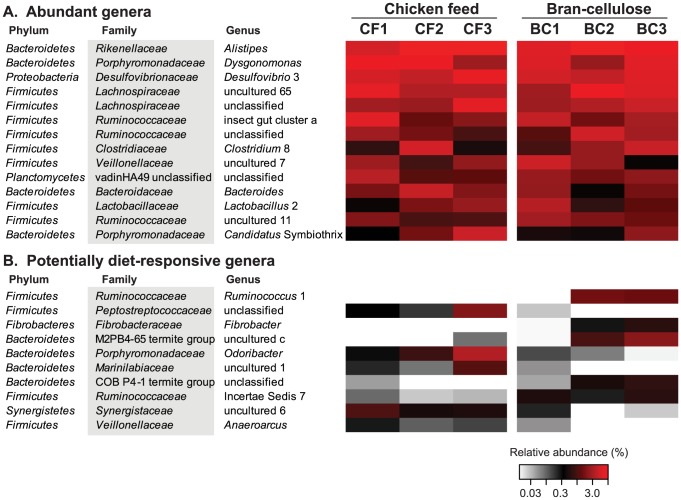
Relative abundance of pyrotag reads from the 15 most-abundant genera (A) and selected candidate genera that showed a potential response to diet (B). The heat map uses a logarithmic scale to increase the visibility of low-abundance groups.

Despite a large amount of variation among the three batches (Table S1), 117 phylotypes were present in all replicate samples of both diets, in each case representing almost half of the sequences (46% in chicken-feed and 49% in bran-cellulose). Many of these shared phylotypes varied strongly in abundance between individual samples, but showed a highly similar distribution at the family level between the diets (Figure 8). Of the 30 families detected, the most abundant were *Ruminococcaceae* (24 phylotypes), *Lachnospiraceae* (21 phylotypes), *Rikenellaceae* (17 phylotypes), and *Porphyromonadaceae* (14 phylotypes). The 15 most-abundant genera together already represented more than half of the sequences in the entire dataset (Figure 9a), with *Alistipes* (6–13% of all sequences) and *Dysgonomonas* (3–10%) being the most abundant. While these and several other highly represented genera had a relatively even distribution, most others varied substantially among batches, irrespective of the diet (Figure 9a, Table S1). None of the taxa showed a clear correlation between abundance and diet; only a few less abundant genera are candidates for a possible diet-related response (Figure 9b).

## Discussion

This is the first comprehensive report exploring the diversity of the bacterial gut microbiota of cockroaches and the impact of diet using a deep sequencing approach. Pyrosequencing analysis identified representatives of nine bacterial phyla that had remained undetected in the previous, clone-based analysis of the bacterial microbiota of *S. lateralis* [2]. Despite numerous reports on the effects of diet on microbial activities in the cockroach gut, our study revealed only a limited impact of diet on bacterial community structure. While a small number of phylotypes present in all diet groups represented the majority of reads in each dataset, others showed a high variability in their distribution that was not correlated with diet. This variability is in agreement with the large individual variations observed for other parameters, such fermentation product patterns or the emission of hydrogen and methane.

The key role of the gut microbiota in the specialization of termites to a wood-feeding lifestyle is evident in the conspicuous expansion in abundance of microbial taxa presumably present already in their omnivorous ancestor, such as those associated with cellulolytic flagellates (in lower termites) [33], [34] or directly implicated in cellulose digestion (in higher termites) [4], [5]. It is not clear whether differences in diet also affect the composition of the gut microbiota in an omnivorous cockroach.

The results of this study indicated that the impact of diet on the gut microbiota of *S. lateralis* is limited. Even after three month of exposure – a time that allowed for at least two moltings – the gut microbiota of the diet groups kept on chicken feed or bran-cellulose comprised only a small fraction of phylotypes that were specific for that particular diet—a proportion likely to decrease if the number of replicates was increased. Also those few phylotypes whose frequency seemed to respond to diet are only of low abundance (Figure 9b). Interestingly, a lineage of uncultured *Fibrobacteres* whose members have been implicated in cellulose digestion in higher termites [4] were detected only in bran-cellulose fed cockroaches.

A relatively small number of bacterial taxa were in common among all diet groups and replicate samples. However, these taxa constitute almost half of all sequences obtained, and although the phylotypes were of variable abundance, they were evenly distributed among diet groups at the family level (Figure 8). Such a dynamic core microbiota has been described in several animals and may be a general feature of the gut ecosystem [35]–[38]. Several studies have differentiated between a taxonomic core (same species present) and a functional core (different species with similar genes, encoding the same metabolic functions) [39], [40]. This core microbiota is thought to provide functional stability and maintain gut homeostasis [39], which is of particular importance for cockroaches that consume a highly variable diet.

Our study revealed significant variation in the community structure of cockroaches, both between individuals (Table 1) and between the replicate batches of different diet groups (Figure 7). The majority of the phylotypes, representing about half of the sequences in the entire dataset, occurred more or less randomly among the different batches, masking the impact of diet in all ordination attempts. That both variability and dietary effects were only visible at the genus level highlights the potential pitfalls of comparing microbial communities only at low taxonomic resolution (phylum or family level) – an unfortunate trend in studies using high-throughput sequencing techniques.

Differences in community structure between individual cockroaches explain the large variations in gut parameters arising from microbial activities, such as the production of short-chain fatty acids, hydrogen, and methane. An increased production of short-chain fatty acids and methane in cockroaches fed high-fiber and cellulose-rich diets, as reported for *P. americana* [21], [22], was not observed in *S. lateralis*. Methane production varied enormously even among cockroaches fed the same diet, and the quantity or pattern of gut fermentation products of cockroaches fed different diets did not change. Although we observed increased colon weight in individuals fed a high-fiber diet (Figure 1), the overall body weights of cockroaches fed different diets did not differ, which is in agreement with a previous study of *P. americana* [22].

Our study shows that cockroaches display substantial individual variation in both gut community structure and related gut parameters when maintained under controlled conditions. Curtis and Sloan [41] postulated that microbial communities of physically identical environments will differ in composition when they are formed from a large and diverse reservoir of microorganisms. In termites, the entire gut community is transmitted through the exchange of droplets of hindgut fluid between nestmates, a social behavior called proctodeal trophallaxis [42]. By contrast, the gut microbiota of non-social cockroaches has to be acquired from the environment – a scenario that is likely to give rise to substantial variation between individuals. Such variation has also been observed in the gut communities of mammals [43]–[45] and may be decided already at an early stage of development [45], [46]. Therefore, dietary effects in the gut community of *S. lateralis* may be masked not only by individual variations but also by unique responses of individual gut communities to the same diet change.

## Supporting Information

Table S1 **Relative read abundance in the pyrotag libraries of the bacterial microbiota in the hindgut of** ***Shelfordella lateralis*** **fed with different diets: soy, chicken feed (CF), bran-cellulose (BC), bran.** Classification results can be displayed for different taxonomic levels (2, phylum; 3, class; 4, order; 5, family; 6, genus).
(XLSX)
